# Psychometric properties of the Iranian version of the thirst distress scale and dietary sodium restriction questionnaire for the elderly with heart failure

**DOI:** 10.22088/cjim.15.3.484

**Published:** 2024-08-01

**Authors:** Khatereh Mohammad Amini, Fatemeh Ghaffari, Ali Pourhabib, Zahra Fotokian, Mohammad Hassan Nadimi Defrazi

**Affiliations:** 1 Student Research Committee, Nursing Care Research Center, Health Research Institute, Babol University of Medical Sciences, Babol, Iran; 2 Nursing Care Research Center, Health Research Institute, Babol University of Medical Sciences, Babol, Iran; 3 Department of Cardiology, Ramsar International Campus, Mazandaran University of Medical Sciences, Sari, Iran

**Keywords:** Thirst distress, Dietary sodium restriction, Heart failure, Elderly, Questionnaire, Validation

## Abstract

**Background:**

Recognizing thirst distress and sodium intake restriction using valid and reliable tools enable evidence-based care, and improve treatment outcomes for the elderly with heart failure (HF). The present study investigated the psychometric properties of the thirst distress scale (TDS-HF) and dietary sodium restriction questionnaire for Iranian's elderly with HF (DSRQ-HF).

**Methods:**

This crossectional study was conducted during 2021-2022. Two hundred and forty elderly people referring to the cardiology clinics and offices in the western region of Mazandaran, Iran were selected by the convenient sampling method. First, the two questionnaires were translated. Then, face, content, and construct validity were assessed. Several indices were used to evaluate, including the chi-square/degree-of-freedom ratio (CMIN/DF), parsimonious normed fit index (PNFI), comparative fit index (CFI), parsimonious comparative fit index (PCFI).

**Results:**

The value of content validity index of all items of the two questionnaires was higher than 0.62. The fit indices, including PCFI=0.594, PNFI=0.582, CMIN/DF=1.987, and CFI=0.979, confirmed the one-factor construct of TDS. PCFI=0.724, PNFI=0.661, CMIN/DF=1.935, ad CFI=0.905, indicated the confirmation of the three-factor construct of DSRQ. The value of Cronbach's alpha of the two questionnaires were 0.86. The value of Ω of the TDS-HF and DSRQ-HF were 0.858, and 0.860, respectively. The value of θ of the TDS-HF and DSRQ-HF were 0.858, and 0.861, representing the suitability of both constructs.

**Conclusion:**

The TDS-HF and DSRQ-HF can be used to measure the psychometric effects of diet therapy and behaviors of the elderly with HF in relation to adherence to diet therapy.

The elderly are susceptible to various chronic diseases, including heart disease (HF) (1). Among cardiovascular problems, HF has a high prevalence in older individuals (2). Based on the European Society of Cardiology, the HF prevalence is 1-3% of the overall population in developed countries. This rate increases to 10% in the over-70 age group (3).The prevalence of HF in Iran is 2%, which is higher than the reported rate worldwide, so it is considered a serious challenge for Iran's health system (4). HF is a complex clinical syndrome in which the heart loses its ability to pump blood properly due to ventricular dysfunction, and the Ejection Fraction (EF) drops to <40% (5-7). Patients with HF may develop pulmonary edema due to excessive fluid intake. Therefore, one of the treatment recommendations for these patients is to limit fluid intake (8, 9).

Restricting fluid intake stimulates the feeling of thirst in patients with HF (9). According to the results of a study, one out of every 5 patients with stable HF suffers from constant thirst, in other words, 19% of patients with HF feel thirsty continuously (10). Thirst distress is the degree of thirst at which a patient with HF cannot resist drinking fluid (9). In a previous study, it was found that during hospitalization, 63% of patients experienced moderate to severe thirst distress, while in an outpatient setting, this was reported by 41% of patients (11). Another study on stable heart failure patients found that 19% of them experienced perceived persistent thirst(12). In this context, TDS was developed for the first time in Germany by Wefer et al. (2020) to measure the extent of thirst distress specifically in HF patients. This 8-item questionnaire is scored on a 5-point Likert scale. The reliability of this instrument was 0.98 (13). 

 Another diet therapy recommended for HF patients is dietary sodium restriction, adherence to which is influenced by health knowledge, age, and the patient's attitude toward diet therapy (14). Dietary sodium restriction means a salt intake of less than 1500 mg per day (14). Research shows that adherence to a low-salt diet is poor in the patients with HF (14-16). In this context, DSRQ was designed and psychometrized by Korkmaz et al. (2020) in Turkey. This 16-item questionnaire, which specifically assesses patients' attitudes, subjective norms, and behaviors in adherence to the restriction of sodium intake(17). 

 Since thirst distress and dietary sodium restriction in the patient's recommended diet can be associated with many consequences, it is necessary to recognize the psychometric consequences of recommended diet therapy early and to know how to adhere to them. Planning and implementing educational and counseling interventions in this regard, especially for elderly patients, can prevent the consequences of the disease and lead to the improvement of the disease and the quality of life of these people (18, 19).

To achieve such goals, variables such as thirst distress and restriction of sodium intake must be measured with valid and reliable instruments appropriate to the cultural structure. The results showed, TDS and DSRQ have not yet been psychometrized in Iran. The lack of access to similar instruments, the simplicity of the items, and the small number of items were one of the reasons for choosing these two instruments for psychometrizing the Iranian elderly with HF. The present study is one of the first studies conducted on HF elderly people in Iran with the aim of investigating the psychometric properties of TDS and DSRQ. Testing the translated questionnaire in urban and rural society and at different educational levels increases the generalizability of the results.

## Methods

This cross-sectional study was conducted during 2021-2022. The study was conducted in two steps: 

**Step 1:** In this step, the TDS and DSRQ were translated using the forward-backward method. In this way, permission was first obtained from the designers of the questionnaire via an email. Then, both instruments were translated from the source language (English) into the target language (Persian).

 This process was based on the translation and equivalence protocol of international quality of life assessment (IQOLA) (20). In the current study, these two instruments were translated into Persian by two translators. Reconciliation and matching of forward translations were performed by the research team. Two other translators independently performed the backward translation (from Persian into English). The final English (backward) translations were reviewed and compared with the original (English) questionnaire by the research team.

**Step 2:** In this step, the psychometric properties of the Persian translations of the TDS and DSRQ were evaluated.

**1) Face validity:** At this stage, 10 HF elderly people completed the Persian version of the questionnaires and were asked to comment on the levels of appropriateness, difficulty, and ambiguity of each item in this version.

**2) Content validity:** To evaluate the validity of the qualitative content, the questionnaire was given to 10 experts (6 nursing faculty, 1doctor of geriatrics, 2 cardiologist, and 1 nutritionist) to comment on wording, grammar, item allocation, and scaling of instruments. The quantitative content validity was evaluated using the content validity ratio (CVR) and content validity index (CVI) methods. To evaluate the CVR, the Lawshe table was used (20). Totally, ten experts who reviewed the content validity were also asked to rate each item's importance on a three-point Likert scale (from "not essential" to "essential"). In the present study, the CVR strict was then calculated, and if it was <0.62, the item was removed (20). Experts (the same ones invited to assess the CVR) were also asked to evaluate the CVI by determining the relevance of each item using a four-point Likert scale ranging from "irrelevant" to "highly relevant".

**3) Construct validity:** A descriptive cross-sectional study was done to assess construct validity. The final version of the questionnaires was administered to 240 elderly people with HF for completion. The demographic characteristics questionnaire consisted of age, educational level, gender, occupational status, marital status, economic status, place of residence, EF, disease type, other diseases, insurance status, and disease duration in addition to TDS and DSRQ.

Two hundred and forty elderly people referring to the cardiology clinics of hospitals and cardiologists' offices in the western region of Mazandaran (Noshahr, Chalus, Tonkabon and Ramsar), Iran were selected by the convenient sampling method during year 2022. After referring to the cardiology clinics of hospitals and cardiologists' offices, the researcher prepared a list of elderly patients with HF, then contacted all individuals on the prepared list, and described the objectives of the study, initial consent to participate. In the present study, we attempted to ensure that the time of the visit to complete the research instruments was when they visited the research sites for follow-up visits. Inclusion criteria included that participants could read and write, had no hearing or vision problems, and had no known psychological disorders (depression, anxiety disorders such as obsessive-compulsive disorder, etc.). Exclusion criteria included incomplete completion of the instruments and unwillingness to cooperate further.

**4) Convergent and discriminant validity:** To evaluate the convergent and discriminant validity of the TDS and DSRQ, the Fornell-Larcker criteria (1981), construct reliability (CR), average variance extracted (AVE), maximum shared squared variance (MSV) and average squared variance (ASV) were applied. If the items of the instrument in a factor have high correlation with each other and are representative of their construct, there is convergent validity, and if the extracted factors are separate, there is discriminant validity (21). AVE>0.5, CR>0.7, and CR>AVE indicate that convergent validity exists, and to confirm discriminant validity, MSV and ASV must be less than AVE (22)

**5) Reliability:** To measure reliability, the TDS and DSRQ were presented to 30 elderly individuals twice, 3 weeks apart. The internal consistency of the TDS and DSRQ constructs in the elderly with HF was calculated using α, Ω, and θ coefficients. The internal stability of the factors is acceptable at more than 0.7 (20). 

**6) Standard error: **In this study, the standard error of the mean (SEM) was examined as one of the indicators of the measurement accuracy and reliability of the instrument. The SEM (SD × √(ICC-1)), minimum detectable change (MDC) (SEM × Z score × √2), minimum important change (MIC) (0.5 × SD of score) were calculated. It is very important that the SEM is minimal because changes greater than SEM are clinically significant (20). In addition, the instrument agreement parameter was evaluated considering MDC and MIC. The instrument agreement parameter is positive when MDC is greater than MIC (20).

**7) Ceiling and floor (C/F) effects:** The C/F effects indicate that the items representing the maximum and minimum intensity of the phenomenon are not included in the questionnaire. The presence of the C/F effect is demonstrated when 15% of the score is obtained at the highest or lowest possible level (23).

**Data Analysis** **:** Data were analyzed using SPSS Version 26. Confirmatory factor analysis (CFA) with maximum likelihood estimation and AMOS 24 were used to validate the constructs of TDS-HF and DSRQ-HF, and JASP was used to evaluate Ω coefficients. Several indices were used to evaluate the model fit indices, including the chi-square/degree-of-freedom ratio (CMIN/DF), parsimonious normed fit index (PNFI), comparative fit index (CFI), parsimonious comparative fit index (PCFI), incremental fit index (IFI), goodness of fit index  (GFI), and root mean square error of approximation (RMSEA) (24).

**Ethical Considerations** **:** The research proposal (code IR.MUBABOL.HRI.REC.1400.243) was approved by the Ethics Committee of Babol University of Medical Sciences. All participants provided written consent and their rights were protected (data were kept confidential and anonymous).

## Results

The mean age of the participants was 69.80 (8.88) years. Disease duration was 11.71 (7.40) years and EF was 26.09٪ (9.56٪), based on echocardiography results. Other demographic and clinical characteristics are presented in table 1. 

**Psychometric results of TDS** **:** Qualitative face validity: In this part, all suggested changes were implemented in the appearance of the items. Quantitative content validity: The results showed that the mean scores of scale-CVI (S-CVI) for all questionnaire items were 0.91. 

**Figure 1:**
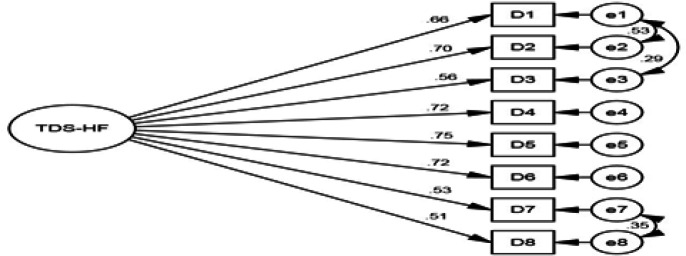
Thirst distress construct in the elderly with HF: confirmatory factor analysis

**Construct validity:** In the CFA, a one-factor model was assessed for the TDS-HF construct, and in this model, all 1-8 items were assigned to one factor (Figure 1). The values of the fit indices (except PNFI and PCFI) of the one-factor model indicate that the proposed model has a poor fit to the data. In the next step, to improve the fit of the one-factor model, the correlation between the covariance errors (e1-e2 , e1-e3, and e7-e8) was plotted and the final modification was made. The fit indices of the modified model including PCFI=0.594, PNFI=0.582, CMIN/DF=1.987, RMSEA=0.064, IFI=0.979, CFI=0.979 and GFI=0.967, showed the confirmation of the one-factor construct of the TDS-HF, which is the appropriate fit of the final model (table 2). 

**Table 1:** Demographic and clinical characteristics of the elderly with HF

**Demographic characteristics**		**Number**	**Percentage**
**Gender**	**Male**	**137**	**57.1**
	**Female**	**103**	**42.9**
**Marital status**	**Single**	**21**	**8.6**
	**Married**	**164**	**68.6**
	**Divorced**	**7**	**2.9**
	**Widowed**	**48**	**20**
**Educational level**	**Literate**	**81**	**34.3**
	**Under diploma**	**89**	**37.1**
	**Diploma**	**48**	**20**
	**Associate degree** **and higher**	**21**	**8.6**
**Occupation**	**Laborer**	**5**	**14.3**
	**Employee**	**21**	**8.6**
	**Self-employment**	**5**	**14.3**
	**Household**	**81**	**34.3**
	**Retired**	**67**	**28.6**
**Income status**	**Insufficient**	**122**	**51.4**
	**Sufficient**	**118**	**48.6**
**Disease** **class**	**I**	**75**	**31.4**
	**I I**	**67**	**28.6**
	**III**	**60**	**25.7**
	**IV**	**5**	**14.3**
**Place of residence**	**Urban**	**118**	**48.6**
	**Rural**	**122**	**51.4**
**Other diseases**	**No**	**14**	**5.7**
	**Yes**	**226**	**94.3**
**Insurance**	**No**	**48**	**20**
	**Yes**	**192**	**80**
**Supplemental insurance**	**No**	**89**	**37.1**
	**Yes**	**151**	**62.9**

**Table 2:** Fit indices of confirmatory factor analysis model of TDS

**Fit indices of CFA model**	**χ** ^2^	**df**	**P-value**	**CMIN/df**	**RMSEA** **(CL90%)**	**PNFI**	**CFI**	**PCFI**	**IFI**	**GFI**
**Pre-modified**	144.968	20	> 0.001	7.248	0.162(0.14-0.19)	0.587	0.841	0.601	0.843	0.864
**Post-modified**	33.773	17	> 0.001	1.987	0.064(0.03-0.09)	0.582	0.979	0.594	0.979	0.967

*Abbreviations; CFA: Confirmatory Factor Analysis; CMIN/DF: Chi-square/degree-of-freedom ratio; RMSEA: Root Mean Square Error of Approximation; PCFI: Parsimonious Comparative Fit Index; GFI: Goodness of Fit Index; PNFI: Parsimonious Normed Fit Index; IFI: Incremental Fit Index; CFI: Comparative Fit Index.

All factor loadings in the modified CFA model were >0.4 and statistically significant. The highest factor loading was related to the item "When I am thirsty, my saliva is very thick" with a factor loading value of 0.747 and the lowest factor loading was related to the item "It is hard for me to overcome the feeling of thirst" with a factor loading value of 0.513 (table 3). 

**Table 3:** Standardized factor loading of the TDS construct

	**Standardized factor loading**	**Standard error**	**Mean**	**Standard deviation**
**1. My thirst bothers me a lot** **.**	0.657	-	3.49	1.07
**2. I am very uncomfortable when I am thirsty.**	0.7.1	0.077	3.45	1.01
**3. When I am thirsty, my mouth is sticky** **.**	0.563	0.093	3.70	1.02
**4. When I am thirsty, my mouth is dry.**	0.718	0.117	3.70	1.03
**5. ** **When I am thirsty, my saliva is very thick.**	0.747	0.121	3.65	1.06
**6. When I drink less water, my thirst gets worse.**	0.721	0.120	3.57	1.06
**7. ** **I am so thirsty that I could drink water uncontrollably.**	0.528	0.125	3.26	1.16
**8. ** **It is hard for me to overcome the feeling of thirst.**	0.513	0.116	3.38	1.08

**Convergent and discriminant validity: **Based on the results, the AVE and CR of the TDS construct were 0.430 and 0.851, respectively, so that CR was >0.7 but AVE was <0.5. Considering that the CR was >0.7; therefore, the convergent validity of the TDS-HF construct was confirmed (table 4).

**Reliability** **:** For the TDS-HF construct, the ICC was 0.989 with a 95% confidence interval. Moreover, there was a correlation between two tests according to the significance of the coefficient (P<0.001), indicating the acceptability and appropriateness of the reliability of the temporal stability or repeatability of the TDS-HF construct over time. TDS scoring: This unidimensional scale consists of 8 items, scored based on a 5-point Likert scale ranging from 1 to 5 ("strongly disagree=1" to "strongly agree=5"). The total score ranges from 8 to 40. A score of 8 indicates no thirst distress and 40 represents severe thirst distress.** Psychometric results of DSRQ**: Qualitative face validity: In this part, all suggested changes were implemented in the appearance of the items.

**Quantitative content validity:** The results showed that the mean scores of the S-CVI for all questionnaire items were 0.94.

**Construct validity:** In the CFA, the three-factor model was investigated. Items 1-6, 7-9 and 10-16 were related to the attitude, subjective norms, and perceived behavioral control, respectively. Considering that RMSEA was <0.1 and CMIN/df was <3, the three-factor model of the DSRQ-HF construct was confirmed. 

The next step was to improve the fit of the three-factor model by plotting the correlation between the covariance errors (e5-e6, e6-e7, e10-e11, e10-e15 and e14-e16) and making the final change. The fit indices of the modified model including PCFI=0.724, PNFI=0.661, CMIN/DF=1.935, RMSEA=0.063, IFI=0.908, CFI=0.905 and GFI=0.912 indicated confirmation of the three-factor construct of DSRQ-HF, representing the appropriate fit of the final model (table 5). 

**Table 4:** Convergent and discriminant validity, internal stability and construct stability of DSR construct in the elderly with HF

**First Order**					**ICC (CL**_90%_ **)**	Ω	θ	α	**Factor**
**CR**	**ASV**	**MSV**	**AVE**						
0.851	-	-		0.430	0.989 (0.973-0.996)	0.854	0.858	0.860	TDS

AVE: Average Variance Extracted; MSV: Maximum shared Squared Variance; ASV: Average Squared Variance; CR: Construct Reliability

**Table 5:** Fit indices of confirmatory factor analysis model of DSRQ

**Fit indices of CFA model**	**χ** ^2^	**df**	**P-value**	**CMIN/df**	**RMSEA(CL90%)**	**PNFI**	**CFI**	**PCFI**	**IFI**	**GFI**
Pre-modified	273.746	101	> 0.001	2.710	0.085(0.07-0.09)	0.626	0.818	0.688	0.821	0.872
Post-modified	185.736	96	> 0.001	1.935	0.063(0.04-0.07)	0.661	0.905	0.724	0.908	0.912

CMIN/DF: Chi-square/degree-of-freedom ratio; RMSEA: Root Mean Square Error of Approximation; PNFI: Parsimonious Normed Fit Index; CFI: Comparative Fit Index; PCFI: Parsimonious Comparative Fit Index; IFI: Incremental Fit Index; GFI: Goodness of Fit Index

**Figure 2:**
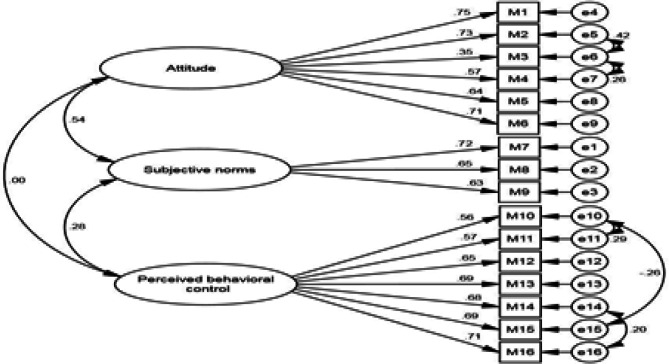
Construct of dietary sodium restriction in the elderly with HF: first-order confirmatory factor analysis

Table 6 and figure 2 illustrate the standardized factor loadings between the items and factors of DSRQ-HF construct in the first-order factor analysis after model modification. All factor loadings in the modified model were >0.4 (21). 

**Convergent and discriminant validity** **:** Based on the first-order CFA results, the AVE values of all factors (0.410-0.445) were >0.5, and the AVE values of each factor were greater than the ASV (0.03-0.18) and MSV (0.07-0.29) values of that factor. Therefore, as shown in table 6, the DSRQ-HF construct has adequate convergent and discriminant validity (table 6).

**Reliability** **:** The ICC value of the DSRQ-HF construct was 0.970 with a 95% confidence interval. Moreover, there was a correlation between two tests according to the significance of the coefficient (P<0.001), indicating the acceptability and appropriateness of the reliability of the temporal stability or repeatability of the DSRQ-HF construct over time (table 7).

The results suggested that the scales of the DSRQ-HF construct had a moderate and poor correlation with each other (p<0.05; r=0.34).

**Standard error** **:** The agreement parameter of the instrument was positively evaluated by considering MDC and MIC for DSRQ and TDS.

**Ceiling and floor effect** **:** In this study, the C/F effect of the DSRQ and TDS constructs was <15%; thus, the C/F effect was not observed and the total scores of both questionnaires were normally distributed.

**DSRQ scoring: **This 16-item questionnaire included 3 subscales of attitude (1-6), subjective norms (7-9), and perceived behavioral control (10-16). The questions in these subscales were scaled from 1 to 5 ("strongly disagree=1" to "strongly agree=5") based on a 5-point Likert scale. Attitude, subjective norm and perceived behavioral control were scored 6-30, 3-15 and 7-35, respectively. A higher score indicated a better situation. However, the questions of perceived behavioral control subscale were reverse coded. A higher score indicates greater obstacles perceived by patients. 

**Table 6:** Standardized factor loading of the DSRQ construct for the elderly with HF

	**Standardized factor loading**	**Standard error**	**Mean**	**Standard deviation**
Attitude			23.21	3.32
It is important for me to follow a low-salt diet.	0.749	-	3.95	0.89
A low-salt diet prevents fluid from accumulating in my body.	0.729	0.159	3.47	0.83
Adhering to a low-salt diet prevents me from becoming bloated.	0.354	0.167	3.58	0.95
A low-salt diet helps me to breathe easier.	0.571	0.161	3.93	0.81
Adhering to a low-salt diet makes me feel better.	0.642	0.163	4.11	0.77
Adhering to a low-salt diet keeps my heart healthy.	0.709	0.155	4.15	0.81
Subjective norms			11.94	1.93
My partner and other family members think that I should adhere to a low-salt diet.	0.717	-	4.07	0.83
I usually want to do what my doctor thinks is right.	0.653	0.228	3.97	0.82
I usually want to do what my partner or family members think is right.	0.626	0.243	3.90	0.91
Perceived behavioral control			18.90	5.07
I don't know any low-salt foods and I don't know how to use them	0.563	-	2.89	1.11
I do not like the taste of low-salt foods.	0.573	0.122	2.74	1.03
I cannot choose low-salt foods in restaurants.	0.652	0.157	2.63	1.04
The restaurants I like do not serve low-salt foods.	0.689	0.153	2.59	0.99
I cannot buy low-salt foods at the supermarket.	0.680	0.159	2.59	1.03
What I like to eat is not low salt.	0.690	0.165	2.64	0.97
I do not have the will to change my diet.	0.712	0.158	2.80	1.13

## Discussion

The aim of the present study was to investigate the psychometric properties of the Iranian version of the TDS-HF and DSRQ-HF. The Iranian version of the TDS-HF and DSRQ-HF has acceptable validity and reliability for the elderly in Iran. These findings align with the results of other studies. The analysis showed a high Cronbach α (0.86) which is comparable with the original scale evaluation (α=0.89), indicating that the translated version of the TDS-HF in Iran maintained strong internal consistency. The translation process was performed linguistically and culturally appropriately.

The present study suggested that the DSRQ is a reliable and valid instrument for assessing the subjective norm, attitude, and internal-external perceived behavioral control of sodium restriction diet among older Iranians with HF. This finding supports the results from the original study (13). The analysis revealed a high Cronbach α score of 0.86, which is comparable with the original scale's α score of 0.92, indicating that the Iranian translation of the DSRQ-HF maintained strong internal consistency.

 The TDS from English to German and the DSRQ from English to Turkish were psychometrized during the translation process, especially for older people with HF. Based on the results of these two studies, the factor loadings of the items of TDS and DSRQ were 0.57-0.86 (17) and 0.67-0.96, respectively (13). In the current study, the highest factor loading on the TDS was associated with the item "When I am thirsty, my saliva is very thick." Thick saliva is one of the symptoms associated with consequences such as xerostomia and stickiness. Therefore, this result could be related to the high prevalence of this symptom in the elderly due to changes related to aging and HF. The item "It is hard for me to overcome the feeling of thirst" accounted for the lowest factor loading. This finding is related to the greater understanding of the consequences of the disease and acceptance of the disease among the elderly in the target group compared to other age groups. Wefer et al. revealed that the highest factor loading (0.86) was associated with the item "It is hard for me to overcome the feeling of thirst" and the lowest factor loading (0.57) was related to the item "When I am thirsty, my mouth is sticky," (13) which are not consistent with the findings of the present study. The reason for the difference in the two studies might be related to the age difference between the participants in the two studies.

 In the DSRQ, the highest factor loading was associated with the item "It is important for me to follow a low-salt diet." Understanding the risk may be the reason for attempting to learn disease management strategies and changing attitudes of older people toward diet therapy and the importance of adherence. The lowest factor loading was related to the item "Adhering to a low-salt diet prevents me from becoming bloated." This finding may be associated with the low level of awareness of the elderly regarding the diet therapy and importance of its adherence.

In the study conducted by Korkmaz et al., the lowest factor loading (0.67) belonged to the item "I do not have the will to change my diet" and the highest factor loading (0.967) belonged to the item "Adhering to a low-salt diet prevents me from becoming bloated" (17). Possible reasons for the inconsistency between these two studies were the different ages of the participants, the level of training provided by the health care providers, and the educational level of the patients in the two studies.

In the ongoing study, in addition to the qualitative method, the content validity index and content validity ratio were also examined to evaluate the content validity of the TDS and DSRQ. These indicators were used to evaluate the wording, item allocation, grammar, and scaling of the instruments, the necessity of the presence of items and the relevance of the items to the desired concept (20). The results demonstrated that the S-CVI was at a high level for all items (25). Wefer et al. and Korkmaz et al. also used qualitative and quantitative methods to assess the content validity of the questionnaire (13, 17).

In the current study, the ICC value showed the acceptability and suitability of the constructs of TDS-HF (0.98) and DSRQ-HF (0.97). The α range for the subjective norm, attitude, and perceived behavioral control dimensions of the DSRQ was 0.78, 0.80 and 0.88, respectively. Furthermore, Wefer et al. and Korkmaz et al. used the internal consistency method to determine the reliability of the TDS and DSRQ (13, 17).

In the studies by Wefer et al. and Korkmaz et al., the reliability of the TDS and DSRQ were in the acceptable range, α=0.89 and α=0.92, respectively (13, 17), which is consistent with the results of the present study. Based on the results of Korkmaz et al., α was 0.97, 0.89, and 0.83 for the dimensions of attitude, subjective norm and perceived behavioral control, respectively (17). In this context, the ICC>0.8 indicates good stability (20). Therefore, the questionnaires in the present study suggested high stability.

In the current study, the standard error rate was positive for both instruments, which is in line with the results of Wefer et al. and Korkmaz et al. (13, 17). In the present study, the skewness and normality of the data of the constructs of the TDS and DSRQ were examined. Based on the results, both tools had a normal distribution. In the studies of Wefer et al. and Korkmaz et al., the data of the constructs of the TDS and DSRQ were normally distributed (17, 13). In the ongoing study, C/F effects were examined to evaluate the discriminative power of the two questionnaires, which represent the accuracy of the present study. However, the studies by Wefer et al. and Korkmaz et al. did not examine the C/F effects (17, 13). The limitations of the ongoing study are: a) not conducting an exploratory factor analysis due to the lack of access to more subjects, b) filling out the questionnaire as a self-report, c) possible influence of participants' physical, mental and psychological condition of the participants in answering the items, d) limiting the sampling to one geographical region of Iran, and e) limiting the sampling to an age group (elderly). In the current study, the results of CFA, α and ICC of the Persian versions of the two questionnaires, TDS and DSRQ, supported the one-factor and three-factor constructs, respectively, and confirmed the validity of these two instruments. The Persian versions of the two questionnaires can be used as valid and reliable instruments to measure the psychometric effects of diet therapy and the attitudes and behaviors of HF older people toward diet therapy. The small number of items, simplicity, and clarity of the items in both questionnaires make them feasible to use during follow-up visits in clinical centers or home visits at the community level by health care providers.
